# Growth and characterization of thorium-doped calcium fluoride single crystals

**DOI:** 10.1038/s41598-023-31045-5

**Published:** 2023-03-08

**Authors:** Kjeld Beeks, Tomas Sikorsky, Veronika Rosecker, Martin Pressler, Fabian Schaden, David Werban, Niyusha Hosseini, Lukas Rudischer, Felix Schneider, Patrick Berwian, Jochen Friedrich, Dieter Hainz, Jan Welch, Johannes H. Sterba, Georgy Kazakov, Thorsten Schumm

**Affiliations:** 1 Laboratory for Ultrafast Microscopy and Electron Scattering (LUMES), Institute of Physics, École Polytechnique Fédérale de Lausanne (EPFL), Station 6, 1015 Lausanne, Switzerland; 2 Institute for Atomic and Subatomic Physics, TU Wien, Stadionallee 2, 1020 Vienna, Austria; 3 Fraunhofer-Institut für Integrierte Systeme und Bauelementetechnologie IISB, Schottkystraße 10, 91058 Erlangen, Germany; 4 TRIGA Center Atominstitut, TU Wien, Stadionallee 2, 1020 Vienna, Austria; 5 CLIP, TRIGA Center Atominstitut TU Wien, Stadionallee 2, 1020 Vienna, Austria

**Keywords:** Chemistry, Materials science, Physics

## Abstract

We have grown $^{232}$Th:CaF$_2$ and $^{229}$Th:CaF$_2$ single crystals for investigations on the VUV laser-accessible first nuclear excited state of $^{229}$Th, with the aim of building a solid-state nuclear clock. To reach high doping concentrations despite the extreme scarcity (and radioactivity) of $^{229}$Th, we have scaled down the crystal volume by a factor 100 compared to established commercial or scientific growth processes. We use the vertical gradient freeze method on 3.2 mm diameter seed single crystals with a 2 mm drilled pocket, filled with a co-precipitated CaF$_2$:ThF$_4$:PbF$_2$ powder in order to grow single crystals. Concentrations of $4\cdot 10^{19}$ cm$^{-3}$ have been realized with $^{232}$Th with good (> 10%) VUV transmission. However, the intrinsic radioactivity of $^{229}$Th drives radio-induced dissociation during growth and radiation damage after solidification. Both lead to a degradation of VUV transmission, currently limiting the $^{229}$Th concentration to $<5\times 10^{17}$ cm$^{-3}$.

## Introduction

The radioisotope thorium-229 has a unique nuclear structure in which the first excited state is long-lived and exceptionally low in energy: few electron volts (eV), instead of the common keV–MeV range for nuclear excited states^1^. The radiative lifetime of this isomeric state ($^{229m}$Th) is expected to exceed 1000 s^2^ for the bare nucleus. Owing to its low energy in the range of electronic shell transitions, an interaction between the nucleus and its chemical environment is expected^3–7^. Studying the interaction of the nucleus with its chemical surrounding presents a unique research opportunity. The $^{229}$Th isomer has attracted many ideas for applications^8^, most of them based on nuclear laser spectroscopy. Our main interest is to perform optical nuclear spectroscopy of vacuum ultraviolet (VUV) transparent single crystals containing $^{229}$Th as a dopant^9^.

The energy of this nuclear isomer state was indirectly measured recently by two independent methods^10,11^ to be $8.15\pm 0.45$ eV (averaged). This corresponds to a wavelength of $152\pm 8$ nm, which is in the VUV range and thus absorbed in air. The measurements of the isomer energy relied on the internal conversion (IC) and gamma emission decay paths from the nucleus, respectively. IC is a common nuclear decay process, where the energy of the excited nucleus is transferred to a shell electron which is ejected if the decay energy exceeds the binding energy. The IC decay channel can have a dramatically different lifetime compared to the radiative decay. The IC lifetime of $7(1)\,\upmu$s was measured for neutral $^{229m}$Th on a metal surface^12^.

To exploit the many prospects of the $^{229}$Th system, internal conversion and other non-radiative decay channels need to be suppressed. For solid-state approaches, this requires the bandgap of the $^{229}$Th-doped crystal material to exceed the isomer excitation energy. Hehlen et al. categorized which large bandgap materials would be suitable, pointing out the relevance of fluoride crystals^13^. Excitation and fluorescence of commercially grown, $^{232}$Th-containing crystals was explored by^14^ (Th:NaYF, Th:YLF, Th:LiCAF, Na$_2$ThF$_6$, Th:LiSAF) to investigate VUV irradiation induced background and optical transparency.

The approach in our laboratory is to use CaF$_2$ crystals with an 11.8 eV direct bandgap^15^. The cutoff of this material is however dominated by a broad indirect exciton bound state at 11.2 eV^16^ which diminishes the VUV transmission for photons with energy above  9.8 eV or wavelength below 126 nm. These exciton states are higher in energy than the 8.15 eV isomer energy and thus non-radiative de-excitation should be prevented.

Crystal doping will ensure a high amount of addressable nuclei, on the order of 10$^{19}$ cm$^{-3}$. The presence of the dopant will however modify the band structure of the host crystal and lead to additional electronic defect states as shown in DFT calculations^9^. Interactions between the nucleus and the local crystal fields will lead to line shifts and broadening^17^.

In this work, we describe the in-house growth and characterization of $^{229}$Th-doped CaF$_2$ single crystals. The detailed growth process is described in “Methods” section. Severe challenges are connected with the inherent radioactivity of the dopant (on the order of 10$^6$ Bq), the required facility and safety measures, its extreme scarcity (milligrams) and general purity requirements of the used materials. To accommodate these, we developed a modified vertical gradient freeze (VGF) apparatus in a radionuclide type C laboratory to grow small-volume crystals ($< 0.1$ cm$^{-3}$) in vacuum with minimal losses. We demonstrate that the doped material maintains VUV transparency in the range of the expected isomer excitation wavelength and investigate the relation between VUV absorption and $^{229/232}$Th doping concentrations. The grown crystals are currently used in several new attempts to excite the $^{229}$Th using X-ray irradiation^18^ and VUV frequency comb irradiation.

## Results

### Vertical gradient freeze growth process

To grow 3.2 mm diameter, 11 mm long crystals, we use a modified vertical gradient freeze method. The method was first developed in 1924 by Stöber^19^. This method was adapted to our needs in cooperation with the Fraunhofer institute for integrated systems and device technology (IISB) to grow crystals with minimal dopant losses^20^. The main advantage of the VGF method over others like Czochralski is that the growth speed is decoupled from the crystal diameter. Very small diameter crystals and hence high (10$^{18}$ cm$^{-3}$) doping concentrations can be realized with the extremely scarce $^{229}$Th isotope while maintaining low growth speed (< 0.5 mm/h) which promotes high quality crystals and improved VUV transparency.

**Figure 1:**
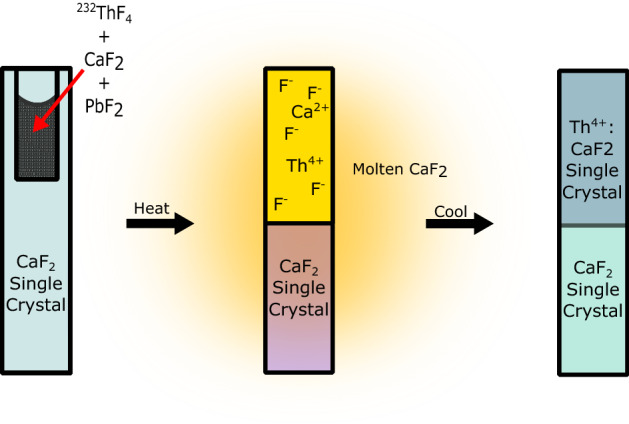
Simplified schematic representation of the vertical gradient freeze method applied to the seed crystal filled with dopant powder. While applying a steep temperature gradient, the top of the crystal melts and becomes a liquid while the bottom remains solid. By slowly cooling and moving the melting interface upwards, a doped single crystal can be grown on the seed crystal.

**Figure 2:**
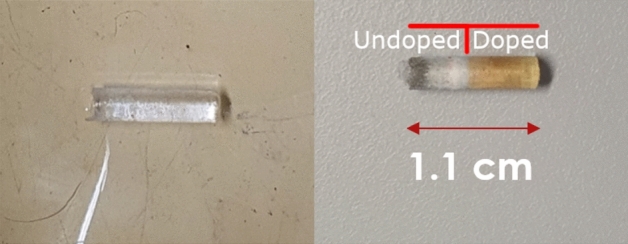
$^{229}$Th:CaF$_2$ single crystal grown with an activity of 1 MBq in the starting material. On the left, the crystal as it was inspected immediately after growth. On the right, the crystal after 3 days. The orange coloring is due to radioactivity-induced agglomerations of defects (F-centers)^21^. The melting boundary can be clearly seen in the color difference, the doped and undoped sides are indicated. The top of the crystal is on the right.

In the vertical gradient freeze method, a steep temperature gradient is slowly driven across the starting growth material (powder and seed) to control the liquid-solid interface, as illustrated in Fig. 1. In our implementation, sub-millimeter control of this interface layer is required, imposed by the small crystal dimensions (3.2 mm diameter, 10  mm long). The crystal growing device is kept under vacuum during the growth process, approximately 10$^{-4}$ mbar at the start of the growth. The vacuum prevents oxidation of the used graphite insulation and CaF$_2$ powder. Inert gases could also be used for this purpose, however achieving a steep temperature gradient (20 K/cm) is then very challenging. An oxygen scavenger is added to eliminate material oxidation prior to the growth process. This growth process succeeded to grow highly radioactive and highly doped ^229^Th:CaF_2_ crystals, a representative image of the crystal is shown in Fig. 2.

### VUV transmission

Good transmission of the obtained crystals in the VUV range around 150 nm is imperative for all attempts to optically manipulate (or detect) the $^{229}$Th isomeric state. We therefore performed a series of characterization experiments described below, with an emphasis on the behavior of the VUV absorption with doping concentration and radioactivity.

Doping Th into the CaF$_2$ matrix should not reduce the bandgap significantly, and thus should not have an appreciable effect on the optical transmission window, as predicted by DFT^9^ and verified experimentally^20^. However, additional electronic states (often referred to as color centers or defect states) can emerge within the bandgap^6^.

**Figure 3:**
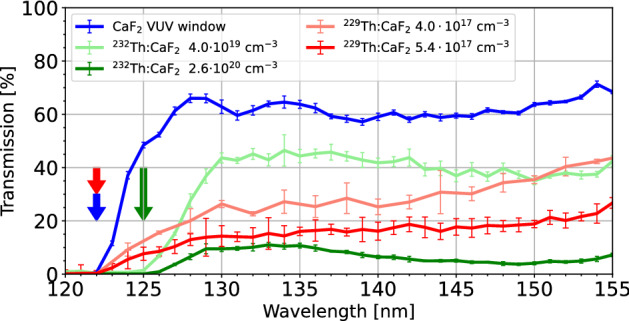
Representative spectral transmissions of ^232^Th:CaF_2_ and ^229^Th:CaF_2_ with different concentrations. A sample of VUV grade CaF_2_, produced and polished by Korth GmbH, is displayed for comparison. The thicknesses of the crystals were respectively 5, 1.7, 1.35, 2.5 and 1 mm. The arrows indicate the approximate transmission edge for each crystal type. A clear shift compared to undoped CaF_2_ in the edge is observed for $^{232}$Th doped crystals but not for $^{229}$Th doped crystals.

To compare measurement results between samples, we account for the thickness of the crystal, surface reflections, and absorption at the surface by normalizing the absorption coefficient to pure (undoped) CaF_2_. This relative absorption coefficient $\mu _{rel}$ then is a measure for the absorption caused by Th doping in the bulk and at the surface. Although generally relevant in CaF_2_, we expect two-photon absorption processes to be negligible in these measurements, due to the very low probe intensities used. It is also assumed that surface quality is similar for all measured crystals. However, this is hard to guarantee due to the hygroscopic nature of calcium fluoride, which leads to water adsorption^22^. The relative absorption coefficient is defined as

1 $$\begin{aligned} \mu _{rel} = \frac{-\log {(T/T_{CaF_2})}}{d}, \end{aligned}$$

where *T* is the sample transmission, $T_{CaF_{2}}$ the transmission of pure CaF_2_ and *d* the thickness of the crystal. We measure the spectral transmission and subsequently calculate the relative absorption of ^229/232^Th:CaF_2_ at 150 nm.

**Figure 4:**
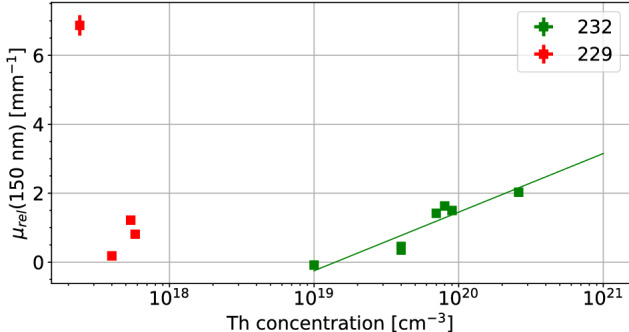
The relative absorption coefficient at 150 nm versus the concentration of Th in the crystal. It can be seen that the absorption of $^{232}$Th doped crystals is dependent on the concentration. The absorption of $^{229}$Th doped crystals is not determined by the concentration of Th due to its low concentration. The highest reached ^229^Th doping concentration was 5.0$\times 10^{18}$ cm$^{-3}$, however this crystal was completely opaque thus was not included. In most cases, the error in both concentration and absorption is negligible. The green guiding line indicates a correlation between the absorption and the concentration.

**Figure 5:**
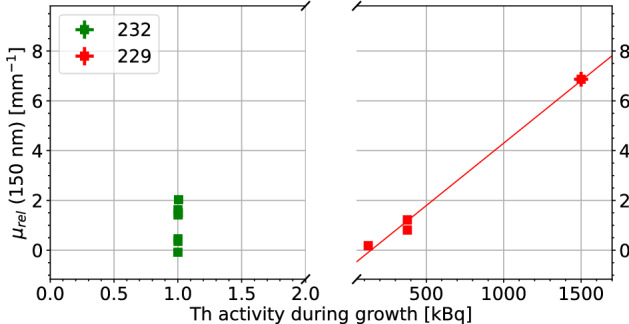
The relative absorption coefficient at 150 nm versus the total activity of Th during growth in the crystal. It can be seen that the absorption of $^{229}$Th doped crystals is dependent on the activity. The absorption of $^{232}$Th doped crystals is not determined by the activity. The total activity of $^{232}$Th doped crystals is dominated by the added 1 kBq $^{229}$Th tracer, but is still too low to have any effect. In most cases, the error in activity and absorption is negligible. The red guiding line indicates a correlation between the absorption and the activity.

Non-normalized transmission measurements for ^232^Th- and ^229^Th-doped crystals can be seen in Fig. 3. Figures 4 and 5 show the relative absorption coefficient at 150 nm, the region of interest for a $^{229}$Th based clock, of all measured crystals as a function of concentration and activity.

From Fig. 3 it can be seen that the grown single crystals have a transmission above 5% around 150 nm, some reaching $\approx$ 40%. In cases where the seed crystal was fully molten during the growth process, we obtain completely VUV-opaque samples (transmission $<0.1\%$, independent of doping concentration). We conjecture that in these cases, a polycrystal is formed, which suppresses VUV transmission due to the presence of grain boundaries that locally increase VUV absorption. Doping concentrations up to 2.6 $\times$ 10^20^ cm$^{-3}$ for ^232^Th and up to 5.4 $\times$ 10$^{17}$ cm$^{-3}$^ 229^Th were reached with good (> 5%) transparency.

The ^232^Th doped crystals often show a broad absorption around 150 nm (see Fig. 3, $^{232}$Th crystals), which we tentatively attribute to Ca metallic particles^23^. Similar absorption bands around 160–170 nm were observed^24^ and attributed to calcium metallic precipitates or colloids incorporated in the CaF$_2$ matrix. These precipitates can form due to crystal damage or a deficiency of fluoride/excess of calcium, which is likely in our crystals due to the present radioactivity. In undoped CaF$_2$ the particles absorb around 160 nm, but the presence of Th will change the refractive index of the crystal thereby possibly changing the absorption wavelength of these particles. Another explanation would be a defect correlated to the $^{232}$Th doping or a concentration-driven local crystal phase change to for example ThCaF_6_^21,25^.

All grown ^232^Th:CaF$_2$ crystals absorb starting from 130 nm and have very low transmission below 125 nm, earlier than the transmission edge of CaF_2_ which starts absorbing at 125 nm and has little transmission below 122 nm. It can be seen that the ^229^Th doped crystals are transparent up until the transmission edge of undoped CaF_2_ as indicated by the arrows. The overall transmission of these crystals is lower but otherwise follows the trend of undoped CaF_2_.

The ^229^Th doped crystals display a different behavior from the ^232^Th doped crystals, which can be clearly seen in Figs. 3, 4 and 5. This was unexpected as it is assumed that different isotopes behave identically concerning their electronic interactions. Most probably, the isotope itself does not change the characteristics but the radioactivity does. The ^229^Th doped crystals only stop transmitting at the bandgap edge of CaF_2_, the 125 nm absorption is not observed. The absorption around 150 nm has also disappeared. The general transmission of the ^229^Th doped crystals is lower, despite the much lower doping concentration.

If we now combine the information contained in all figures, a few observations can be made:

- Crystals grown with increasing activity have an activity-dependent broadband VUV absorption, independent of Th concentration. This is clearly seen in Figs. 4 and 5. The ^232^Th doped crystals are radioactive due to the small amounts of added ^229^Th in combination with the weak activity of ^232^Th. The $^{232}$Th activity of the crystal with the highest ^232^Th doping concentration is 5.5 Bq.
- Thorium doping with low activity creates a concentration-dependent weak absorption around 150 nm and strong absorption around 125 nm. This is mainly visible for crystals with doping concentration of > 4$\times$ 10^19^ cm$^{-3}$.

The thorium-related absorption around 125 nm does not seem to be present in crystals grown with high activities. It is conjectured, that the thorium is in a different electronic state, either oxidation state or paired with a defect, if a crystal is grown in the presence of high radioactivity. Because of this change in electronic state, the absorption around 122 nm disappears. The hypothesis connected with this observation is that the radioactivity induces loss of fluoride which produces non-stoichiometric, or fluoride-deficient crystals. These CaF_2_ crystals thus have a non unity ratio between Ca and F_2_, which changes the electronic configuration of the Ca and the Th thereby changing the absorption profile.

### Cherenkov radiation

A second characterization was performed where the radioluminescence of the $^{229}$Th:CaF_2_ crystal, grown with 100 kBq of ^229^Th, was measured in the VUV spectral range. This crystal was chosen due to its high concentration and transparency. The inherent radioactivity of the $^{229}$Th produces two main types of luminescence: Luminescence of CaF$_2$ by creating electron-hole pairs which form self-trapped excitons (STE)^26^ and Cherenkov radiation through beta emission with energies larger than 158 keV^27^. Stellmer et al. measured both these emission types by using undoped CaF$_2$ and a solid $^{233}$U sample. The Cherenkov radiation dominates the low wavelength region up until 200 nm. Above 200 nm, the luminescence of the STEs dominate. Both can produce a background for further experiments and thus merit characterization.

**Figure 6:**
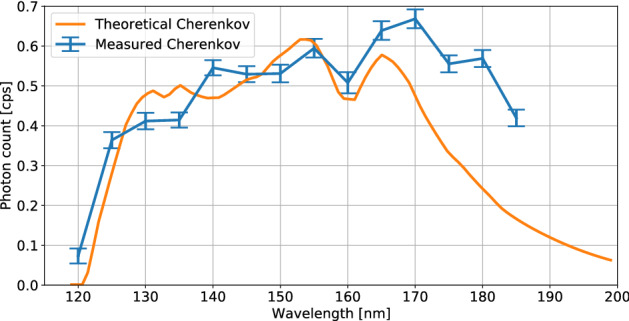
The detected VUV spectrum of a $^{229}$Th:CaF$_2$ crystal compared to a theoretically computed Cherenkov spectrum that takes into account the spectral efficiency of the system. See “Methods” for a description of the measurement system.

The Cherenkov background at 150 nm was measured to be 0.53 cps, see Fig. 6. The calculation was slightly scaled to experimental results (see below for calculated discrepancy) to accentuate the differences between the curves. A 1 mm slit size was used for the measurement. The maximum count rate was 0.7 cps. The absorption edge of CaF$_2$ below 122 nm can be seen, as well as loss of efficiency of the PMT and grating for higher wavelengths. The measured transmission of the crystal was used to calculate the theoretical Cherenkov spectrum. The sharp peaks at low wavelength in the theoretical spectrum are artefacts due to lines in the D_2_ lamp used to measure the transmission of this crystal.

As can be calculated^27^, every single decay event (1 Bq) of $^{229}$Th produces 0.1 Cherenkov photons per second at 150 nm in a 1 nm bin. Considering 9.4 kBq (the amount of nuclei visible through a narrow slit) of $^{229}$Th, and 0.05% total efficiency of the spectrometer system, the Cherenkov flux at 150 nm would be 0.47 cps. The small discrepancy between prediction and measurement can be due to the (lack of) accuracy in the measured amount of $^{229}$Th in the crystal, or the characterization of the numerous experimental efficiencies. In this calculation the following efficiencies were considered: the measured transmission of the crystal, the efficiency of the grating and the efficiency of the PMT with its MgF_2_ window.

The calculated Cherenkov spectrum does not fully reproduce the measured values for higher wavelengths > 170 nm. This could be caused by measurement errors in the transmission of the crystal, which is used in the calculation of the spectrum. The mismatch can also be VUV luminescence of color centers induced by the radioactivity: radioluminescence. It is known that due to the scavenger, all crystals are slightly contaminated with Pb which has a luminescence peak at 180 nm^28^. A more detailed investigation on the luminescence properties (radioluminescence and VUV-induced) is under way with the aim to identify contaminants as well as the additional electronic levels (defect centers) connected to Th doping in different charge states.

## Discussion

We have grown highly doped ($5.4\times 10^{17}$ cm$^{-3}$) and highly active (1 MBq) $^{229}$Th:CaF$_2$ crystals with low absorption ($\mu$ = 0.9 mm$^{-1}$ or 20% transmission at 150 nm and 2 mm thickness) and compared them to grown $^{232}$Th:CaF$_2$. This was done by careful co-precipitation of ThF$_4$ and PbF$_2$ and mixing with CaF$_2$ to form a powder of growth material. The powder was then placed in a specially machined, millimeter-scale single CaF$_2$ seed crystal which was used to grow a single Th:CaF$_2$ crystal on top of the seed crystal using the vertical gradient freeze method. At high activities, these crystals seem to lose VUV transmission. We hypothesise that the differences in transmission for the ^232^Th and ^229^Th doped CaF_2_ crystals are due to radiolysis induced fluoride deficiency.

The strongest evidence for a fluoride deficiency in the ^229^Th:CaF_2_ crystals was the transmission of the low doped ^229^Th:CaF_2_ crystal, grown under an activity of 1 MBq (see Fig. 2). A thin slice taken from the unmolten (but not completely undoped, see “Methods”) side of this crystal, was measured to be completely VUV opaque. At first the suspected cause was radiation damage. However, annealing to 600 $^\circ \hbox {C}$ did not decrease the VUV absorption of the undoped and doped parts, whereas it should remove radiation damage^29^. The annealing did however remove the orange color of the doped parts in Fig. 2, which is the healing of F centers. Not responding to annealing indicated that the VUV opaqueness was not radiation-induced damage in the crystal after growth. Another observation hinting towards fluoride-deficiency was that when growing CaF_2_ crystals in vacuum (doped or not) outgassing of F_2_ was measured using a mass spectrometer.

**Figure 7:**
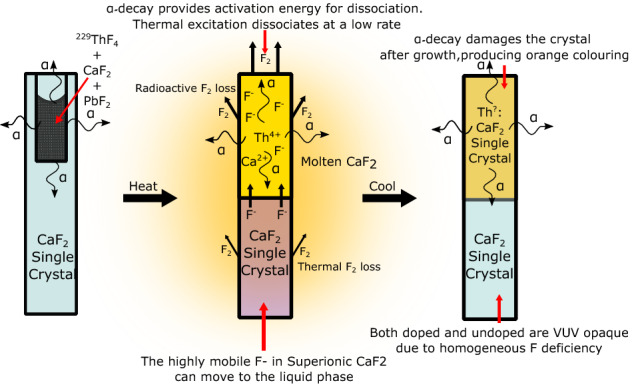
Schematic representation of the radioactively induced loss of fluoride during the growth process (compare to Fig. 1), taking the 1 MBq ^229^Th crystal as an example (see Fig. 2). First the crystal is filled with the radioactive powder. During growth, a part of the crystal is molten and a part of the crystal is in the superionic state. In the liquid, the ^229^Th is dissolved and the $\alpha$-decay locally provides the energy to dissociate CaF_2_, producing F_2_ that is pumped away. The superionic crystal has extremely mobile F$^{-}$ atoms which will diffuse to the liquid phase, supplying it with more F$^{-}$. The resulting crystal will have a homogeneous deficiency of F due to its high mobility during growth, but only a partial doping of Th due to its low mobility during growth. As is shown in “Methods”, only a small fraction of the Th penetrates the unmolten crystal.

Our conjecture of the process that leads to the fluoride deficient crystals is depicted and detailed in Fig. 7. By growing CaF_2_ with radioactive materials, the loss of fluoride in the liquid phase is enhanced through radiolysis and non-stoichiometric crystals are produced. The dissociation of fluoride compounds to produce gaseous F_2_ through radioactivity, radiolysis, has been observed in CaF_2_^30^. Any radiolysis before growth does not affect the end result because the loss of fluoride (which in air will be replaced by oxygen) is mitigated through the use of oxygen scavengers. The nuclear chemical reaction describing the radiolysis process is

2 $$\begin{aligned} \text {CaF}_2 \xrightarrow []{\alpha \text { 5 MeV}} \text {Ca(s)} + \text {F}_{2}(g), \end{aligned}$$

where the metallic Ca is dissolved in the solid phase, and F_2_ leaves the liquid melt as a gas. The energy for a chemical reaction is $\approx$ eV, the energy of $\alpha$-decays is $\approx$ MeV. Because of this, every single $\alpha$-decay could drive many chemical reactions. In a conservative estimate, if 1% of all reactions with an $\alpha$ particle drive radiolysis, which costs 10 eV per reaction, then 5000 dissociation events can take place per $\alpha$-decay. At an activity of 1 MBq of ^229^Th this would mean at least $5\times 10^9$ radiolysis events per second. In a growing cycle where a part of the crystal is molten for 22 h, there are 2 $\times 10^{15}$ radiolysis events as compared to the $\approx 10^{21}$ F atoms in the crystal. This would lead to a defect concentration of 4 $\times 10^{16}$ cm$^{-3}$, which could be visible in an absorption measurement.

Below the melting temperature, between 1097 and 1177 $^\circ \hbox {C}$^31,32^, the CaF$_2$ undergoes a phase transition into the superionic state. In the superionic state, the fluorides are highly mobile inside the crystal Ca$^+$ matrix, which remains relatively immobile. The observation that the unmolten part is fluoride deficient means that either the superionic unmolten bottom feeds F$^{-}$ to the melt, where the radioactivity induces further dissociation, or after (or during) solidification of the melt the entire crystal is superionic and the fluorides are homogeneously redistributed. Both options result in an opaqueness of the unmolten part and deficiency throughout the crystal. The crystal compensates for F$^{-}$ loss by producing Ca metallic colloids which increase the VUV absorption throughout the crystal. Still, even at 1 MBq total doping activity, a transmission of several percent at 150 nm remains. In the future, attempts will be made to regain the undoped CaF$_2$ transmission profile by re-feeding fluoride.

The thorium-related spectral absorption feature at 125 nm decreased in strength for radioactive crystals and the same crystals showed unidentified emission features in the Cherenkov spectrum at higher wavelengths. It is hypothesized that the spectral features observed in radioactive crystals are caused by a change in the electronic configuration around the Th dopant due to a fluoride deficiency. The addition of fluoride to the radioactive crystal is expected to alter the electronic configuration, resulting in absorption spectra similar to those of non-radioactive crystals. Density Functional Theory (DFT) calculations have predicted that the ground state of Thorium is Th^4+^ with two interstitial F^-^ ions as charge compensation^33^. Our experimental results are consistent with the prediction, evidenced by an absorption at 125 nm in the ground state (non-radioactive crystals)^6^ that is absent in the radioactive crystals, indicating that the electronic configuration of the dopant has changed. The highly active $^{229}$Th:CaF$_2$ crystals emit Cherenkov radiation which was quantified in this work, as it will constitute a constant background for the future nuclear spectroscopy of $^{229}$Th in the crystal matrix. To prove that the material is suitable for nuclear spectroscopy, the Thorium-229 in the CaF_2_ must be exposed to VUV or x-ray irradiation, followed by observation of the radiative de-excitation. These experiments are currently underway. Further characterization of the material is needed to determine the local electronic structure of the thorium dopant. The discrepancies between radioactive and non-radioactive crystals remain unclear and are being investigated.

## Methods

The growth and characterization of radioactive doped Th:CaF_2_ crystals is a complicated process. A detailed step by step description of each process and details on setups can be found in^21^. Here we present a summary of all necessary steps.

### Growth recipe

First, the starting material is prepared using coprecipitation of ThF_4_ with CaF_2_. This process combines the materials and allows us to handle the extremely small amounts of ^229^ThF_4_ by combining it with CaF_2_. Following is a description of the used crystal growing device, the temperature calibration process and growth process. Finally a characterisation of the doping efficiency and homogeneity is provided.

#### Preparation of ^229^ThF_4_:PbF_2_:CaF_2_ growth material

In an exemplary fashion, the following describes the preparation of 45 mg of $^{229}$ThF$_4$:PbF$_2$:CaF$_2$ powder for growing 3 crystals (15 mg each) with 3.2(1) mm diameter and 11(1) mm length. PbF$_2$ acts as a scavenger for oxygen removal (see below) and as a carrier that facilitates the handling of the minuscule (micrograms) amounts of $^{229}$ThF$_4$ during the wet chemistry preparation.

$^{229}$Th (7.9 MBq, Oak Ridge National Laboratory, in dried nitrate form) was dissolved in 0.1 M HNO$_3$ Suprapure grade (Sigma Aldrich) prior to use. All reagents CaF$_2$ (Alfa Aesar), Pb(NO$_3$)$_2$ (Sigma Aldrich), PbF$_2$ (Alfa Aesar), 40% HF (Sigma Aldrich) were purchased from commercial suppliers in trace metal grade and were used as received. Water was purified in-house by triple distillation. Using higher quality CaF$_2$ powder increased the VUV transmission of the grown crystals.

In a centrifugation vial, lead(II) nitrate (2.9 mg) was added to and dissolved in a solution of $^{229}$Th in 0.1 M HNO$_3$ (9 mL, 5.5 MBq). Subsequently, $^{229}$ThF$_4$:PbF$_2$ was precipitated by addition of hydrofluoric acid (40%, 1 mL). A white precipitate appeared immediately and was allowed to rest overnight. The supernatant was carefully removed using a pipette after centrifugation and the precipitate was washed with triple distilled water (2 mL, 6 times). After the fourth washing step, the supernatant was tested for remaining free fluoride ions by adding a small portion of an aqueous solution of CaCl$_2$. No appearance of any white material confirmed the absence of free fluoride ions, and two additional washing steps were performed.

The $^{229}$ThF$_4$:PbF$_2$ was then poured into an aluminum container to avoid powder sticking to the wall, reducing losses when the powder is transferred into the crystal growing apparatus later. A small portion of water was added and the whole was then dried in an oven at 80 $^\circ \hbox {C}$ until weight was constant (4 days). Then CaF$_2$ (28.3 mg) was added to the $^{229}$ThF$_4$:PbF$_2$, mixed thoroughly and measured via $\gamma$-spectroscopy. Due to losses during the process 4.7 MBq out of 5.8 MBq $^{229}$Th were obtained as usable powder. The powder was then combined with a previous batch of $^{229}$ThF$_4$:PbF$_2$:CaF$_2$ (15 mg, containing 0.3 MBq $^{229}$Th) to give a total amount of 45 mg $^{229}$ThF$_4$:PbF$_2$:CaF$_2$ with a weight ratio of 0.33:1:14 and a total activity of 5.0 MBq $^{229}$Th.

#### Preparation of ^232^ThF_4_:PbF_2_:CaF_2_ growth material

For calibration purposes, process optimization, and many measurements, which do not probe nuclear properties (e.g. VUV transmission measurements), commercially available $^{232}$Th can be used as a proxy. The preparation was equivalent to powder containing $^{229}$Th.

#### Crystal growing device and temperature cycle

**Figure 8:**
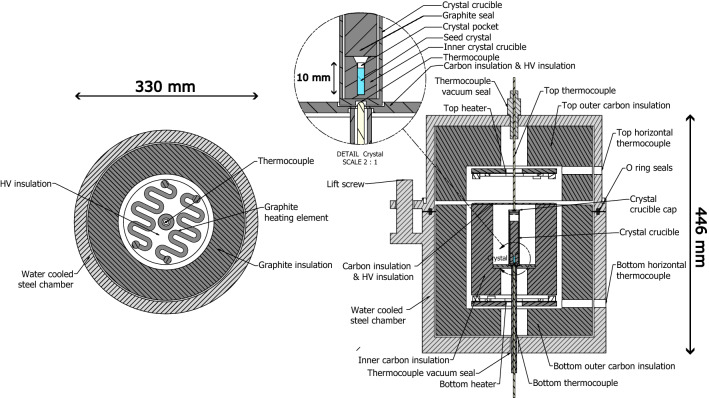
Crystal growing device used to grow 3.2 mm diameter crystals. On the left side is a horizontal cut displaying the graphite heating element with the isolated thermocouple through the center. The right image displays a vertical cut that shows all of the main components of the vertical gradient freeze growing apparatus and zooms into the crystal location.

In the vertical gradient freeze method a steep temperature gradient is required to grow a doped crystal on top of a seed, as depicted in Fig. 1. The vacuum vessel containing heaters and graphite insulation is depicted in Fig. 8. The gradient is realized by electronically controlling the currents in two ohmic heaters on top of and below the crucible containing starting powder (described above) and seed.

A stationary graphite crucible is filled with a CaF$_2$ seed together with the starting material in a pocket drilled in the seed. Seed crystals of 5 mm diameter (with different, single crystal orientations) were purchased from Korth, Matek, and Alkor. Hyperion Optics milled the seeds down from 5 to 3.2 mm diameter and drilled a 2 mm diameter hole (5 mm deep) into the top, to accommodate the doping material. A temperature gradient of $\approx$ 20 $^\circ \hbox {C}$/cm should be around the melting temperature of the chosen material, which is 1418 $^\circ \hbox {C}$ for CaF$_{2}$. In this way, the powder in the pocket of the seed crystal can be molten together with the top part of the seed. Due to the rather steep gradient the bottom of the seed does not melt. The freezing interface is then slowly moved upward such that the melt can crystallize on top of the seed crystal, and thus grow a single crystal following the orientation of the seed.

The pocket is filled with the co-precipitated growth material described in previous sections. Special care was taken to use metallic funnels just as the containers to avoid material losses due to electrostatic adsorption to the wall. The pocket within the seed ensures minimal losses and easy handling of the crystals before starting the VGF process. The filled seed is placed into the VGF furnace (Fig. 8) inside of a carbon crucible. The phase diagram of CaF$_2$ and ThF$_4$ at low vacuum has not been measured to our knowledge, but we observe evaporation. Fluorine was detected by using a Pfeiffer quadrupole mass spectrometer and traces of radioactive Th and U in the graphite show that these partly evaporate during growth. The volume above the seed in which material can be evaporated is small in our setup. We observed that this small volume increases doping efficiency as opposed to a larger volume or direct connection to the vacuum pump. We hypothesize that when the vapor is actively pumped away more of the material is evaporated which reduces doping concentration.

The seed is then grown to a single crystal: Two ohmic carbon heaters (see left side of Fig. 8) are used to create the steep temperature gradient over the crystal, partially melting it. Short-term temperature stability is maintained with short horizontal alsint-insulated thermocouples close to the heaters and absolute calibration is done with long vertical thermocouples close to the crucible. The thermocouples are platinum/rhodium (Pt30Rh–Pt6Rh) with an alsint casing. The graphite thermal isolation and alsint isolation of the thermocouples must not touch, since at high temperatures these two chemically react, which slowly degrades the casing of the thermocouples which can create vacuum leaks. The vacuum seals for the thermocouples are made of FKM, which is able to withstand both the low pressure fluorine atmosphere and the high temperatures which are present at the ends of the thermocouple. Water is circulated through the steel vacuum chamber to provide cooling.

The temperature cycle of the growing process is divided into five sections: (1) 18 h of heating up the system, outgassing, and restoring pressure, (2) 6 h of scavenging oxygen through reaction with PbF$_2$, (3) 22 h of melting the top half of the crystal and also slowly freezing it, (4) 18 h of annealing the crystal, (5) 14 h of cooling down. A vacuum of at least 10$^{-4}$ mbar is obtained before growth. During growth (especially during the first section) the pressure can go up to 10$^{-2}$ mbar. The complete growth process typically takes 3 days.

Due to the radioactive nature of the dopant, special security measures are implemented in the growth process. The entire process is performed in radionuclide type C laboratories, when opening the vacuum chamber personal protective equipment is worn. On the pre-pump, carbon filters are installed to absorb any evaporated material. The dopant material also is absorbed into the graphite insulation, which becomes radioactive after several growth cycles. In every growth, the insulation absorbs and also releases some dopant; cross-contamination of dopants was observed in a pure CaF_2_ crystal, grown after growth of a radioactively doped crystal. The growth of undoped crystals can be used to reduce cross-contaminations by absorbing them, we observe a reduction of roughly a factor 10 per growth process.

One important aspect in growing CaF$_2$ crystals is the probability of incorporating oxygen, especially at higher temperatures^34,35^. Oxygen contamination is known to reduce the transparency of CaF$_2$, especially in the VUV region. At elevated temperatures, the carbon crucible should react with any background gaseous O$_2$ and H_2_O to form CO in the system. The H$_2$O that is adsorbed on the surface of the crystal powder, however, will first react with the CaF_2_. The H_2_O will react with the CaF$_2$ to CaO (T_melt_ = 2613 $^\circ \hbox {C}$^36^) and HF.

To mitigate this, oxygen scavengers are used: Fluoride compounds (PbF_2_ T_melt_ = 830 $^\circ \hbox {C}$^36^) in the powder that before melting CaF_2_ react preferentially with oxygen and water to volatile oxygen containing compounds (PbO T_melt_ = 887 $^\circ \hbox {C}$^36^) which are evaporated at higher temperatures and transported away such that the oxygen is replaced by fluoride.

#### Doping efficiency and homogeneity

Before and after growth, $^{229}$Th-doped crystals are measured on a $\gamma$-spectrometer in a reproducible geometry (in the crucible) to determine the efficiency and homogeneity of the doping process by comparing the intensity of the 193 keV $\gamma$-line of the ^229^Th decay. We observe a 20–30% doping efficiency of the starting material where we attribute the losses to evaporation. The highest doping concentration realized so-far was with a $^{229}$Th activity of 1.5 MBq (5.4 $\times 10^{17}$ nuclei) in the starting material, reaching a maximum concentration of 5.0 $\times 10^{18}$ cm$^{-3}$. The doped part of this crystal was completely opaque.

The doping concentration is homogeneous over the molten part of the crystal and decays over 2-3 mm in the interface to the not molten seed. For this investigation, a crystal doped with $^{232}$Th, spiked with 1 kBq $^{229}$Th, was grown. After growth the crystal was cut into 1 mm disks and each disk was measured in a reproducible geometry in the gamma-detector. Again, the 193 keV line activity was detected and from this the concentration was calculated by measuring a reference sample. The seed crystal was molten to a depth of around 6 mm, in which the concentration is approximately homogeneous. Beyond, we observe diffusion of dopant into the not molten part on a length scale of about 2 mm, until no doping can be detected in the not molten region of the seed crystal. More details can be found in^21^.

### VUV transmission methods

In order to measure the VUV transmission, the crystals were cut and polished. Cutting was done using a Wiretec DWS100 with a 0.08 mm diamond coated wire. Facets cut with the wire saw were flat enough to be polished in a 1-step process. Polishing was done with a Buehler polishing machine and a Buehler SiC P4000 Silicon Carbide polishing paper. As CaF_2_ is hygroscopic and adsorbed water decreases transmisson, the polishing paper was wetted with isopropanol instead of water^37^. All operations were performed with personal protective equipment in a radionuclide type C lab in well ventilated boxes.

Transmission measurements were performed using a dedicated setup. The light of a Hamamatsu L15094 D_2_ lamp is focused with a toroidal mirror onto the entrance slit of a McPherson 234/302 monochromator. The light is separated into its spectral components by the grating and is focused onto the exit slit. By rotating the grating the exit wavelength can be selected. The exit slit cuts out a small portion of the spectrum effectively creating a narrow wavelength light source with a linewidth down to 0.1 nm. The linewidth can be changed by changing the entrance/exit slit width (0.01–2.50 mm). This light travels through the crystal, and is recorded by a Hamamatsu R6835 head-on CsI photomultiplier tube (PMT) which is mounted close to the crystal.

Although conceptually simple, measuring a wavelength-dependent absolute absorption is burdened with several experimental challenges. These are connected with geometrical changes in the beam paths due to the presence of the sample (beam shifts and astigmatism), strong spectral intensity modulations and overall intensity instabilities in the VUV source (deuterium lamp). These lead to an overall systematic error on the following transmission measurements of ± 5%.

### VUV luminescence methods

For the luminescence measurements, the chosen 100 kBq ^229^Th:CaF_2_ crystal was placed in the focal point of the entrance of a 234/302 McPherson spectrometer. The slit in front of the crystal was set to an opening of 1 mm, corresponding to 4 nm resolution. Any radioluminescence was then spectrally resolved and imaged on a Hamamatsu R7639 PMT cooled to −30 $^\circ \hbox {C}$. The PMT is cooled to reduce dark noise to 0.5 cps, otherwise Cherenkov radiation could not be detected. A shutter was used to take continuous dark measurements while the signal was integrated for 9 h per wavelength setting.

## Data Availability

VUV absorption and luminescence data together with analysis scripts can be found on the Zenodo depository^38^ under the name “Growth and characterization of thorium doped single crystals” at https://doi.org/10.5281/zenodo.7341378.
